# P295: Endoscopes’ maintenance: results from an audit conducted in Beni-Messous University Hospital Algiers in 2012

**DOI:** 10.1186/2047-2994-2-S1-P295

**Published:** 2013-06-20

**Authors:** G Brahimi, R Belkaid, A Larinouna, C Lafer, A Soukehal

**Affiliations:** 1 Department of Epidemiology and Preventive Medicine, CHU Béni-Messous, Algiers, Algeria

## Introduction

Medical devices used in endoscopy should be disinfected according to best practices to eliminate microorganisms on their surfaces.

## Objectives

Evaluate the practical disinfection of endoscopes.

## Methods

The audit was conducted from 03 to 19 January 2012, in the services using endoscopy. Data collection was done through observation and interviews of staff responsible for the disinfection of endoscopes from a predetermined rubric. Entry and data analysis were performed on Epi-info6.

## Results

7 services use Endoscopy (Otorhinolaryngology, Pulmonary and Allergy Medicine, Allergy, General surgery, *Pulmonary function tests*, CPPA, Internal Medicine, Pediatric). 15 people responsible for the disinfection of endoscopes were audited. 66.7% are nursinggraduates, 21.4% are instrumentalists. More than 50% have an average experience of 14 ± 9 years. Disinfection of endoscopes every morning before first use is achieved in 40% of cases. The seal is tested in 74% of cases.

The second cleaning is never done. The final rinsing takes place with tapwater, drying with a sterile field in 40% and by blowing compressed air in 20% of cases.

The storage isn’t daily cleaned and disinfected. Periodic maintenance of the unit is done neither by the manufacturer nor the distributor.

## Conclusion

This audit revealed the lack of necessary materials, and non-compliance with the disinfection protocol of endoscopes in some services and the absence of microbiological testing.

## Competing interests

None declared

